# Prevalence and complications of diabetes mellitus in Northern Africa, a systematic review

**DOI:** 10.1186/1471-2458-13-387

**Published:** 2013-04-25

**Authors:** Manouk Bos, Charles Agyemang

**Affiliations:** 1 Academic Medical Centre, University of Amsterdam, PO Box 22660, Amsterdam, The Netherlands

**Keywords:** Type 2 diabetes, Diabetes complications, North Africa

## Abstract

**Background:**

Diabetes is increasingly becoming a major chronic disease burden all over the world. This requires a shift in healthcare priorities and up-to-date data on the epidemiology and impact of diabetes in all regions of the world to help plan and prioritize health programs. We systematically reviewed the literature on diabetes prevalence and its complications in the UN sub region of Northern Africa including Morocco, Algeria, Tunisia, Libya, Egypt, Sudan, South Sudan and Western Sahara.

**Methods:**

A systematic literature review of papers published on diabetes prevalence and complications in North Africa from January 1990 to July 2012. Literature searches were conducted using electronic databases.

**Results:**

Diabetes prevalence ranged from 2.6% in rural Sudan to 20.0% in urban Egypt. Diabetes prevalence was significantly higher in urban areas than in rural areas. Undiagnosed diabetes is common in Northern Africa with a prevalence ranging from 18% to 75%. The prevalence of chronic diabetes complications ranged from 8.1% to 41.5% for retinopathy, 21% to 22% for albuminuria, 6.7% to 46.3% for nephropathy and 21.9% to 60% for neuropathy.

**Conclusions:**

Diabetes is an important and common health problem in Northern Africa. Variations in prevalence of diabetes between individual countries are observed. Chronic complications of diabetes are common. Urgent measures are needed to prevent diabetes and its related complications in Northern Africa.

## Background

Type 2 diabetes is increasingly becoming a major chronic disease health burden in Africa. In 2011, about 14 million individuals were estimated to have diabetes in Africa, and this is expected to rise to 28 million by 2030 [1].

The highest increase is seen in urban areas [2]. Changing patterns of diet, physical activity, and ageing populations are thought to be the major drivers of the increasing prevalence of diabetes in Africa [3]. Cheap availability of high-fat and high-energy food in combination with less physical activity has led to the increasing prevalence of obesity [4]. Obesity can cause impaired glucose tolerance, which can lead to increased susceptibility to diabetes manifestation.

In 2011, a systematic review summarized the prevalence and outcomes of diabetes in the Sub-Saharan Africa [5]. It confirmed the increase in diabetes prevalence and its complications in the Sub-Saharan Africa. The authors concluded that when effective interventions are implemented in the near-future it may be possible to avert the diabetes burden in this region. This requires a shift in global health priorities and therefore more evidence on prevalence and areas of intervention is needed.

While a systematic review in the Sub-Saharan Africa shows a clear increase in the prevalence of diabetes, the diabetes situation in Northern Africa has not yet been systematically assessed. Mapping the situation can be an important base for policy on diabetes prevention and treatment. Therefore the main aim of this review was to fill this knowledge gap by providing an up-to-date overview on diabetes prevalence and related microvascular complications including retinopathy, nephropathy, neuropathy and microalbuminuria in North Africa.

## Methods

### Search strategy for identification of studies

We conducted a systematic review of all papers published on diabetes in adults in North Africa between January 1990 to July 2012 and available in PUBMED database. North Africa in this article is defined as the UN sub region and includes Morocco, Algeria, Tunisia, Libya, Egypt, Sudan, South Sudan and Western Sahara [6]. Studies were included if they were based on adults aged ≥18 years, were carried out in the UN sub region of North Africa, assessed prevalence of diabetes and related microvascular complications, and if the sample size was ≥50 participants. The search was limited to studies done in humans and no language restrictions were used.

### Search strategy for identification of studies on prevalence of diabetes

Combined keyword search on PUBMED identified 1037 articles of which 962 were excluded because studies were conducted outside the region of interest, described diabetes pathogenesis, included genetic or microbiological research, reviewed another disease, used data based on the analysis of patients records, or were case reports (see Additional file 1 for a description of the review and Additional file 2 for the flow-chart). Of the remaining seventy-four articles after removal of duplicates, twenty-one papers were reviewed for prevalence of diabetes. Two papers were manually included after examining the references of included articles. In total, twelve studies met the inclusion criteria and were included for data on prevalence of type 2 diabetes in North Africa [7-18].

### Search strategy for identification of studies on diabetes complications

Fifty-three papers were reviewed for data on microvascular (retinopathy, nephrology, neuropathy and micoalbuminuria) diabetes complications of which forty-six were excluded because they were based on management and screening options, and conditions such as periodontal problems, mental health problems, or their sample size was too small (i.e. n<50). Two studies were manually included after examining the references of included articles. In total, nine articles met the inclusion criteria and were included for data on microvascular diabetic complications [19-27].

## Results

### Prevalence of diabetes, undiagnosed diabetes, impaired glucose tolerance and impaired fasting glucose in Northern Africa

Twelve community-based studies provided data on diabetes prevalence and all studies were based on random samples. Six of the eight countries in the Northern African region had data on prevalence of diabetes in the last twenty years (Table 1). The prevalence of diabetes varied across Northern African countries ranging from 2.6% in rural Sudan [17] to 20% in urban Egypt [10]. Ten studies distinguished between urban and rural diabetes prevalence and all of these studies found a higher prevalence in urban areas than in rural areas [7,9-13,15-18].

**Table 1:** Prevalence of type 2 diabetes in North Africa 1995-2012

	**Author**	**Sample size (participation rate %) +Methods**	**Age range**	**site**	**Methods**	**Prevalence (%) (95%CI)**			**Prevalence undiagnosed diabetes (% of all diabetics)**	**Prevalence IGT (%)(95%CI)**	**Prevalence IFG (%)**		**% Obese (BMI **≥** 30) (95% CI)**	
						**Males**	**Females**	**Total**			**Males**	**Females**	**Males**	**Females**
Algeria	Malek 2001 [7]	1457 (90) Random sample	30-64	Urban and rural	OGTT (WHO 85)	8.6	7.8	8.2 (6.8-9.6) Urban: 9.7 Rural: 7.3	4 8	7.1 (5.8-8.4)		-	-	-
	Latifa 2007 [8]	805 (72) Random sample	≥20	Urban	FBG (WHO)	15.7	16.5	16.1	-	-	-		10.5	27.9
	Zaoui 2007 [9]	7656 Random sample	≥20		FBG (WHO 85)	15.8 Urban: 17.1 Rural: 14.1	7.5 Urban: 7.1 Rural: 7.8	10.5 Urban: 10.8 Rural: 10.1	-	-			43.2 Urban: 56.7 Rural: 27.2	37.1 Urban: 51.8 Rural: 20.0
Egypt	Herman 1995 [10]	4620 (41–85) Random sample	≥20	Urban and Rural	RPG+FBG (WHO 85)	Urban, higher SES: 12.9 Urban, lower SES: 7.0 Rural: 2.7	Urban, higher SES: 8.1 Urban, lower SES: 10.0 Rural: 2.1	Urban, higher SES: 20.0 Urban. lower SES: 13.5 Rural: 4.9	Urban, higher SES: 50 Urban, lower SES: 38 Rural: 51	Urban, higher SES: 8.6 Urban, lower SES: 5.4 Rural: 31.1			Urban, higher SES: 56 Urban, lower SES: 19 Rural: 6	Urban higher SES: 45 Urban, lower SES: 64 Rural: 25
Libya	Kadiki 2001 [11]	868 (86.6)	≥20	Urban and Rural	OGTT (WHO 98 ADA 97)	16.3 (14.5-18.3)	13 (10.0-16.1)	14.1 (10.9-17.1) Urban: 14.5 Rural: 13.5	38 Urban: 18 Rural: 49	8.5 (5.8-11.3) Urban: 5.8 Rural: 11.8			with diabetes: 19.5 with IGT: 11.8	
Morocco	Tazi 2003 [12]	1662 (96.4) Random sample	≥20	Urban and Rural	FBG (ADA 97)	6.6 (4.7-8.6)	6.6 (5.1-8.1)	6.6 (5.4-7.8) Urban: 9.0 (7.1-10.8) Rural 4.4 (3.0-5.8)					7.2 (5.2-9.1)	19.1 (16.5-21.6)
Tunisia	Bougerra 2007 [13]	1735 (96.4) Random sample	≥19	Urban and Rural	FBG (WHO 80 ADA 97)	9.5 Urban: 12.3 Rural: 5.6	10.1 Urban: 12.5 Rural: 5.9	9.9	75	-	5.3 Urban: 5.5 Rural: 4.9	5.1 Urban: 4.8 Rural: 5.7	BMI ≥25: 5.4	BMI ≥25: 4.2
	Ghannem 1992 [14]	555 (76) Random sample	≥20	semi-Urban	OGTT (WHO 85)	6.3	6.6	6,5	39	-			12	26
	Ghari 2002 [15]	692 (69) Random sample	35-50	Urban and Rural	FBG (ADA 97)	6.1 Urban: 9.3 Rural: 2.0	8.9 Urban: 10.4 Rural: 4.5	7.2 Urban: 9.9 Rural: 3.4	31	-				37
	Elasmi 2002 [16]	2483 (99.6) Random sample	35-70	Urban and Rural	FBG (ADA 97)	16 Urban: 17 Rural: 11	Urban: 15 Rural: 15						19 Urban: 19 Rural:: 11	46 Urban: 47 Rural:: 37
	Elbagir 1996 [17]	1284 (not reported) Random sample	≥25	Urban and Rural	OGTT (WHO 85)	3.5	34	3.42 (2.4-4.4) Urban: 3.87 (2.6-5.2) Rural: 2.62 (1.2-4.1)	64 Urban: 63 Rural: 67	2.88 (2.0-3.8) Urban: 3.27 (2.1-4.5) Rural: 2.18 (0.8-3.5)				
	Elbagir 1998 [18]	724 (not reported) sample	≥25	Urban and Rural	OGTT (WHO 85)	Urban: 15.8 (9.2-22.4) Rural: 2.8 (2.1-7.7)	Urban: 10.7 (6.4-15.0) Rural: 8.3 (3.5-13.1)	8.25 (6.3-10.3) Urban: 9.54 (6.9-12.2) Rural: 6.08 (3.2-9.0)	62 Urban: 36 Rural: 50	7.87 (5.8-9.8) Urban: 8.03 (5.5-10.5) Rural: 7.60 (4.4-10.8)			BMI≥27: 24	BMI≥25: 45

*OGTT*: Oral Glucose Tolerance Test *FBG*: Fasting Blood Glucose *RPG*: Random Plasma Glucose Test WHO 80/85/89: WHO Health Organozation 1985/98/99 diagnostic criteria ADA 97/04 America Diabetes Association 1997 diagnostic criteria *IFG:* Impaired Fasting Glucose *IGT*: Impaired Glucose Tolerance *SES*: Socioeconomic Status.

Eight studies reported on undiagnosed diabetes [7,10,11,13-15,17,18]. The prevalence of undiagnosed diabetes ranged from 18% in urban Libya [11] to 75% in Tunisia [13].

Five studies reported on impaired glucose tolerance [7,10,11,17,18]. Four of these studies distinguished between urban and rural areas. Two of these four studies found a higher prevalence of impaired glucose tolerance in rural areas than in urban areas [10,11]. The highest prevalence of impaired glucose tolerance (13.1%) was found in rural Egypt [10]; and the lowest prevalence was found in rural Sudan with a prevalence of 2.2% [17]. One study in Tunisia reported on impaired fasting glucose and the prevalence was 5.3% in men and 5.1% in women, respectively [13].

### Prevalence of obesity

Ten studies also assessed the prevalence of obesity among their study populations [8-16,18]. Six of the eight studies that distinguished between males and females found a significantly higher prevalence of overweight/obesity in females than in males in Algeria, Egypt, Morocco, Tunisia and Sudan [8,10,12,14,16,18]. The obesity prevalence ranged from 56% in men with higher socioeconomic status in urban Egypt to 6% in men in rural Egypt [10]. The study in Sudan also found a high prevalence of obesity, with a prevalence of 45% in women, but in this study obesity was defined as a BMI ≥25 [18]. The lowest obesity prevalence was found in one study conducted in Southern Tunisia. Although this study in Tunisia defined obesity as a BMI ≥25, still they found the lowest prevalence of 5.4% in men and 4.2% in women [13]. By contrast, another study in Greater Tunis region found a high prevalence of obesity with 19% in men and 46% in women, respectively [16].

### Complications of diabetes in Northern Africa

Nine studies [19-27] on the prevalence of complications of diabetes were reviewed (Table 2). The prevalence of retinopathy ranged from 8.1% in Tunisia [26] to 41.5% in Egypt [20]. Albuminuria prevalence ranged from 21% in Egypt [20] to 22% in Sudan [24]; nephropathy ranged from 6.7% in hospital outpatient clinics in Egypt [20] to 46.3% in hospital inpatients in Egypt [21]. The prevalence of diabetic neuropathy ranged from 21.9% in hospital outpatient clinics [20] to 60% in hospital inpatient clinics in Egypt [21]. High prevalence of neuropathy was also found in Sudan with a prevalence of 31.5% in hospital inpatient clinics to 36.7% in outpatient clinics, respectively [23,24].

**Table 2:** Chronic complication of diabetes in North Africa 1995-2012

**Complication**	**Author (year)**	**Location**	**Sample**	**Setting**	**Type of Diabetes**	**Prevalence % (95% CI)**
Retinopathy	Macky 2011 [19]	Egypt	1325	hospital outpatient clinic	Mixed	20.5 (16.3-26.4)
	Herman 1998 [20]	Egypt	1451	outpatients clinic	Mixed	41.5 (35.8-47.2)
	Hamed 2008 [21]	Egypt	8O	hospital inpatient clinic	Mixed	20
	Kadiki 1999 [22]	Egypt	960	hospital outpatient clinic	Type 2	30.5
	Emahdi 1991 [23]	Sudan	413	hospital inpatient clinic	Type 2	17.4
	Elbagir 1995 [24]	Sudan	91	hospital outpatient clinic	Mixed	43
	Harzallah 2006 [26]	Tunisia	370	hospital inpatient and outpatient clinic	Mixed	8.1
	Ayed(1993) [27]	Tunisia	400	Hospital outpatient clinic	Mixed	37.5
Albuminuria	Herman 1998 [20]	Egypt	1451	outpatient clinic	Mixed	21.0 (15.4-26.5)
	Elbagir 1995 [24]	Sudan	128	Hospital outpatient clinic	Mixed	Proteinuria: 22
Nephropathy	Herman 1998 [20]	Egypt	1451	outpatient clinic	Mixed	6.7 (3.3-7.0)
	Hamed 2008 [21]	Egypt	80	Hospital inpatient clinics	Mixed	46.3
	Kadiki 1999 [22]	Libya	960	Hospital outpatient clinics	Type 2	25.2
	Elmahdi 1991 [23]	Sudan	413	hospital inpatient clinic	Type 2	9.2
	Harzallah 2006 [26]	Tunisia	370	hospital inpatient and outpatient CliniC	Mixed	13.1
Neuropathy	Herman 1998 [20]	Egypt	1451	outpatient clinic	Mixed	21.9 (17.7-26 0)
	Hamed 2008 [21]	Egypt	SO	hospital inpatient	Mixed	60
	Kadiki [22]	Libya	960	hospital outpatient	Type 2	45 7
	Elmadi 1991 [23]	Sudan	413	hospital inpatient	Type 2	31.5
	Elmagir.199S [24]	Sudan	128	hospital outpatient	Mixed	36.7
	Ahmed 1993 [25]	Sudan	120	hospital inpatient	Mixed	40.0
	Harzallah 2006 [26]	Tunisia	370	hospital inpatient and outpatient clinic	Mixed	24.3

## Discussion

### Key findings

This review shows that diabetes is a common health problem in Northern Africa. We observed a variation in diabetes prevalence between different countries in Northern Africa. Almost all the studies, which distinguished between urban and rural areas observed a higher diabetes prevalence in urban than in rural areas. Complications of diabetes are common in Northern Africa.

### Limitations

This review has some limitations. The availability of data on the prevalence of diabetes in Northern Africa over the past twenty years is limited and therefore it was impossible to describe the trends of diabetes prevalence over time. In the reviewed studies, different methods were used to diagnose diabetes. This could have led to differences in diabetes prevalence between Northern African countries. This also makes it impossible to carry out meta-analysis of the results. In addition, the reviewed studies were conducted in different years, varying from 1990 to 2012. In order to make an accurate estimate of prevalence differences between countries, it would be ideal to compare studies conducted in the same period of time. This was impossible because of the limited availability of data on prevalence of diabetes in Northern Africa. Despite these limitations, this current review still provides valuable information about one of the important chronic disease conditions and its complications in North Africa.

### Discussion of key findings

Increasing urbanization and life expectancy are expected to lead to an increase in number of people with diabetes. The rising levels of urbanization are likely to lead to a high prevalence of obesity due to lifestyle changes such as changing diet and physical activity patterns. The Mediterranean diet patterns are considered as healthy and associated with decreased morbidity and mortality [28]. This diet is shifting towards a more westernized diet, which is associated with increased prevalence of diet-related conditions such as obesity. Dietary energy, measured in kilocalories per capita per day has been steadily increasing in all the North African countries [28]. Among the North African countries, the lowest intake of fat is found in Egypt while the highest intake is found in Libya [28]. For Libya, this finding is in line with the high prevalence of diabetes. The fat intake in Egypt, however, contrasts the reported high prevalence of diabetes. This suggests that other factors might contribute to diabetes prevalence in Egypt. In one study, the prevalence of diabetes in people with higher socioeconomic status was the highest in the whole of Northern Africa. In addition, higher socioeconomic status was associated with decreased physical activity and increased prevalence of obesity [10]. This seems to suggest that the fat intake might differ across socio-economic and cultural groups in Egypt.

The differences in diabetes prevalence between urban and rural areas in most Northern African countries indicate that urbanization is a major factor for the increasing prevalence of diabetes in North Africa [12]. The minor rural–urban difference in diabetes prevalence in Libya (13.5% in rural areas versus 14.5% in urban areas) may be due to a couple of reasons. First of all, there have been rapid socioeconomic changes in Libya since the discovery of oil in 1961. Only little differences in housing, lifestyle and obesity between urban- and rural areas exist and essential food items are subsidized by the governance [11].

Because of the limited availability of data it is hard to describe trends of diabetes prevalence over time. Nonetheless, one study in this review provided data on diabetes prevalence in 1980 and in 1996 in Tunisia. These data show a clear increase in the prevalence of diabetes between 1980 and 1996. For example, in 1980 the prevalence rates of diabetes in men were 2.3% and 4.3% in rural and urban Tunisia, respectively. In 1996, however, the prevalence of diabetes was 4.0% in rural men and 11.4% in urban men. Similar increases were also observed in women [13].

Diabetes prevalence in Northern Africa is at an intermediate to high level compared to Sub-Saharan Africa. Diabetes prevalence in urban areas is 10-12% in Kenya and 10% in Zimbabwe. The prevalence of diabetes in Sudan and Morocco, which are countries with the lowest diabetes prevalence of this review, are in the same prevalence range as most countries in Sub-Saharan Africa [5].

Compared with diabetes prevalence found in the Arab States of the Gulf, diabetes prevalence in Northern Africa is intermediate. For example, high prevalence rates of diabetes were observed in Lebanon (13.1%), Jordan (13.4%), Kuwait (14.8%) and Bahrain (25.5%) [29-32]. In addition, a systematic review of studies from the Gulf observed a prevalence of impaired glucose tolerance varying from 10% to 20% [33].

The prevalence of undiagnosed diabetes is higher than 50% in four of the eight studies which included data on this subject [10,13,17,18]. This high prevalence of undiagnosed diabetes is common in low- and middle-income countries, [17] and is found in urban as well as in rural areas. This reflects on insufficient national diabetes programs in many low- and middle-income countries. Due to a lack of knowledge on diabetes, people do not recognize its symptoms and this may lead to delay in diagnosis.

This review shows a high prevalence of chronic diabetes complications. The prevalence of microvascular complications was higher than previously observed in African countries [24]. It is very likely that poor metabolic control contributes to the higher prevalence of chronic diabetes complications since hyperglycaemia is significantly related to complications [23]. Poor metabolic controlled patients are common. In one study in Sudan, about 45% of the patients had poor control and that this was mainly due to non-compliance with diet, drugs and lack of education [23]. Most patients were unaware of their complications and a high percentage of patients with severe complications were never seen by a specialist before [24]. In Tunisia, patients were screened for diabetes complications at the time of diagnosis. The high prevalence of retinopathy, which is the most specific complication of hyperglycaemia, suggests a delay between the onset of diabetes and the time of diagnosis [26]. Patients lack of knowledge on diabetes complications may also contribute to the high rates of complications. In one study in Egypt, about 80% of the patients lacked the knowledge about the ocular hazards of diabetes [19].

The findings of this review have important policy implications for North Africa. Overall, less attention has been given to non-communicable diseases in North African countries by health planners as in other low- and middle- income countries [11]. Consequently, obesity, a major risk factor for diabetes is culturally prized in some social groups especially among those with less education [34]. In Libya, a national diabetes program has been ongoing since 1984. Diagnostic facilities, insulin and hypoglycemic medicine are available for free. Unfortunately, there are still many deficiencies in this diabetic program [11].

## Conclusions

This review suggests that diabetes and related complications are common problem in Northern Africa and suggest an urgent need for action to prevent diabetes and its related sequelae in this world region. The situation still requires more up-to-date research to help guide clinical and the preventive efforts.

## Competing interests

The authors declare that they have no competing interests.

## Authors’ contributions

MB and CA conceived the idea, developed and refined the methodological approach. MB carried out the literature search, extracted the data and wrote the first draft of the manuscript. MB and CA contributed to the interpretation of the results. All authors read and approved the final manuscript.

## Pre-publication history

The pre-publication history for this paper can be accessed here:

http://www.biomedcentral.com/1471-2458/13/387/prepub

## Supplementary Material

Additional file 1 Keyword search terms. Click here for file

Additional file 2 Study selection with flow diagram based on the PRISMA 2009 guidelines. Click here for file
