# Supplementary material for: Prevalence and complications of diabetes mellitus in Northern Africa, a systematic review
Source: BMC Public Health. 2013 Apr 25;13:387. doi: 10.1186/1471-2458-13-387 (PMC3646695; doi:10.1186/1471-2458-13-387)
Supplement: Additional file 1 — Keyword search terms. [file 1471-2458-13-387-S1.doc]

# Additional file 1 Keyword search terms

| **Search** | **Search Terms** | **Hits** | |
| --- | --- | --- | --- |
| **1** | Diabetes OR Diabetes Mellitus | 408377 | |
| **2** | "Africa, Northern"[Mesh] OR ("africa"[All Fields] AND "north"[All Fields]) OR "French Speaking Africa"[All Fields] OR "French-Speaking Africa"[All Fields] OR "Maghreb"[All Fields] OR ("algeria"[MeSH Terms] OR "algeria"[All Fields]) OR ("egypt"[MeSH Terms] OR "egypt"[All Fields]) OR ("libya"[MeSH Terms] OR "libya"[All Fields]) OR ("morocco"[MeSH Terms] OR "morocco"[All Fields]) OR ("tunisia"[MeSH Terms] OR "tunisia"[All Fields]) OR ("sudan"[MeSH Terms] OR "sudan"[All Fields]) OR (South[All Fields] AND ("sudan"[MeSH Terms] OR "sudan"[All Fields])) OR (Western[All Fields] AND ("africa, northern"[MeSH Terms] OR ("africa"[All Fields] AND "northern"[All Fields]) OR "northern africa"[All Fields] OR "sahara"[All Fields])) | 65723 | |
| **3** | #1 AND #2 | 1564 | |
| **4** | #3 Limits: 1990/01/01 to 2012/07/01 and studies done in Humans | 1037 |  |
