# Supplementary material for: Prevalence and complications of diabetes mellitus in Northern Africa, a systematic review
Source: BMC Public Health. 2013 Apr 25;13:387. doi: 10.1186/1471-2458-13-387 (PMC3646695; doi:10.1186/1471-2458-13-387)
Supplement: Additional file 2 — Study selection with flow diagram based on the PRISMA 2009 guidelines. [file 1471-2458-13-387-S2.docx]

# Additional file 2. Study selection with flow diagram based on the PRISMA 2009 guidelines

Records identified through database searching
1037

*Adapted From:* Moher D, Liberati A, Tetzlaff J, Altman DG, The PRISMA Group (2009). *Preferred Reporting Items* for *Systematic* Reviews and *Meta- Analyses:* The PRISMA Statement. PLoS Med 6(6): e1000097. doi:10.1371/journal.pmed1000097

10

7

Records identified by hand-searching of references of included articles
2

Records identified by hand-searching of references of included articles
2

Full-text articles excluded, with reasons
46
N≤50,
study not conducted in UN subregion of Northern Africa
Study conducted in children
Diabetes prevalence and/or prevalence of diabetes complications not explored

Full-text articles assessed for eligibility on diabetes **complications**
53

Studies included in systematic review
9

Studies eligible for inclusion
9

Studies included in systematic review
12

Studies eligible for inclusion
12

## Eligibility

## Included

Full-text articles excluded, with reasons
11
N≤50,
study not conducted in UN subregion of Northern Africa,
Study conducted in children
Diabetes prevalence and/or prevalence of diabetes complications not explored

Full-text articles assessed for eligibility on diabetes **prevalence**
21

## Screening

Records screened
1036

Records excluded
962

Records after duplicates removed
1036

## Identification
